# An integrated metabolome and transcriptome analysis of the *Hibiscus syriacus* L. petals reveal the molecular mechanisms of anthocyanin accumulation

**DOI:** 10.3389/fgene.2022.995748

**Published:** 2022-09-05

**Authors:** Xiaohong Wang, Lihua Li, Caixian Liu, Minhuan Zhang, Yafeng Wen

**Affiliations:** ^1^ Central South University of Forestry and Technology, Changsha, China; ^2^ Hunan Big Data Engineering Technology Research Center of Natural Protected Areas Landscape Resources, Changsha, China

**Keywords:** anthocyanidins, bluish-purple petal color, glycosylation, Hibiscus spp., hormone signaling, MBW-complex, Sharon rose

## Abstract

*Hibiscus syriacus* L. var. Shigyoku is a new double-flowered bluish-purple variety in China that changes color during flower development from bluish-purple to light purple. There is limited information on the anthocyanin accumulation patterns and associated transcriptome signatures in Shigyoku from D1 (bud) to open flower (D3). Here, we employed a combined transcriptome and metabolome approach to understanding the mechanism of this color change. Our results demonstrate that cyanidins, pelargonidins, delphinidins, petunidins, peonidins, and malvidins were differentially accumulated in Shigyoku petals. The anthocyanin biosynthesis started in D1, was significantly upregulated in D2 (semi-open flower), and reduced in D3. However, malvidins, pelargonidins, and peonidins could be associated with the bluish-purple coloration on D2. Their reduced accumulation in D3 imparted the light purple coloration to Shigyoku petals on D3. Significant contributions in the color change could be associated with the expression changes in anthocyanin biosynthesis genes i.e., LARs, ANSs, DFRs, UGT79B1, C3’Hs, 3ATs, and BZ1s. The UFGTs were associated with the higher accumulation of glycosylated anthocyanins in D2 and D3. Furthermore, the changes in the expressions of the MYB and bHLH transcription factors were consistent with the anthocyanin accumulation. Finally, we discussed the possible roles of Jasmonic acid, auxin, and gibberellic acid signaling in regulating the MBW complex. Taken together, we conclude that *H. syriacus* petal coloration is associated with anthocyanin biosynthesis genes, the MBW complex, and phytohormone signaling.

## Introduction

*Hibiscus syriacus* L., a member of the *Malvaceae* family, is commonly known as Sharon rose. It is an ornamental flowering plant, and more than 350 varieties are being grown worldwide (Magdalita and San Pascual, 2020). Its flowers are used in salads and are known to possess biological functions against a wide range of human diseases (Kim et al., 2022). It is widely used as a Chinese medicinal plant because of its pharmacological activities i.e., antifungal, antifertility, antihypertensive, anti-inflammatory, and antibacterial activities (Punasiya et al., 2014). The *H. syriacus* varieties are categorized based on two factors i.e., the number of petals and petal color. It has a long flowering period that extends from May to October, but the blooming period of a single flower is usually 1 day. The flower color changes throughout the flower development i.e., from pod to full bloom. This color-changing characteristic is particularly prominent in a new double-flowered cultivar, “*H. syriacus* Shigyoku” (Shigyoku thereafter). Its flowers show bluish-purple petals when semi-opened and turn purple when completely open (full bloom). There is a need to explore the petal color transition mechanism of *H. syriacus* for breeding flowers of different color intensities to target different consumers. Additionally, petal color is an important biological factor that attracts pollinators (Whitney and Glover, 2007).

*Hibiscus syriacus* flowers contain nearly 40 different anthocyanin components. Flowers from different varieties in this species produce different amounts of cyanidin, delphinidin, procyanidin, peonidin, pelargonidin, petunidin, and malvidin to produce different colorations (Zhang et al., 2022). The different shades of color such as lavender, blue, red, and purple colors are produced *via* anthocyanin pigments (Zhao et al., 2012). The three main types of anthocyanins are distinguished by the number of hydroxyl groups on their B-ring: anthocyanins generated from pelargonidin (orange or red; one hydroxyl group), cyanidin (magenta; two hydroxyl groups), and delphinidin (containing malvidin imparting blue or purple; three hydroxyl groups) (Ng et al., 2018). The functional groups present on the anthocyanins skeletons improves the color of the compounds as well as the plant tissue where they are synthesized. For example, the red and purple petals of *H. syriacus* flowers contained more anthocyanins and were substantially hydroxylated and partially hydroxyl methoxylated, resulting in a deeper hue of the petal cell vacuole (Alappat and Alappat, 2020). The reduction/loss of anthocyanins also contributes to color transition in flowering plants e.g., *Ipomoea purpurea* (Zufall and Rausher, 2004), *Linanthus parryae* (Schemske and Bierzychudek, 2001), and *Primula vulgaris* (Li et al., 2020). Moreover, the mutations in the coding sequences of the anthocyanin biosynthesis pathway (ABP) genes have also been implicated in the evolutionary transition in floral color (Streisfeld et al., 2011).

Anthocyanins biosynthesis in plants takes place through ABP, which is a component of the flavonoid biosynthesis pathway (Liu et al., 2018). Upstream of the flavonoid pathway, the phenylpropanoid pathway has the early ABP genes such as phenylalanine ammonia-lyase (PAL). Together with PAL, the ABP genes in dicots are divided into two groups i.e., early biosynthetic genes (EBGs) and late biosynthetic genes (LBGs) (Weiss, 2000). The EBGs include chalcone synthase (CHS), chalcone isomerase (CHI), and flavanone 3-hydroxylases (F3H), which are the common flavonoid biosynthesis pathway genes and affect downstream flavonoids. Whereas the LBGs include flavonoid 3′-hydroxylase (F3′H), flavonoid 3′,5′-hydroxylases (F3′5′H), dihydroflavonol 4-reductase (DFR), leucoanthocyanidin dioxygenase (ANS), and UFGTs (Tanaka et al., 2008). Several internal factors e.g., co-pigments, cell shape, pH, phytohormones (Griesbach, 2010) play an important role in flower coloration. The transcriptional control of the ABP is associated with the MBW (MYB, bHLH, and WD40 TFs). Two components of this complex i.e., MYB (Docimo et al., 2016) and bHLH (XIE et al., 2012) are positive regulators of ABP genes and in most, cases their expressions are specific to the pigmented tissues. On the other hand, WD40 plays similar roles in both pigmented and non-pigmented tissues. Nevertheless, WD40 stabilizes the MBW complex (Li, 2014). Studies have shown that the MBW complex is influenced by plant hormone signals. Especially, the activation/deactivation by Jasmonic acid (JA), auxin, and gibberellic acid (GA) is relatively well established (LaFountain and Yuan, 2021). Although, these mechanisms of anthocyanin biosynthesis, transcriptional control, and regulatory signals are well explored in a range of flowering plants, but how these pathways control the anthocyanin accumulation and petal coloration in different *H. syriacus* (in general) varieties especially Shigyoku (specifically) is yet to be explored.

Developments in transcriptomics and metabolomics have geared up the exploration of complex pathways that regulated traits e.g., ABP. We adopted a combined metabolome and transcriptome analyses to explore the ABP in Shigyoku petals. We specially looked into the ABP and flavonoid biosynthesis pathway. We found that the flavonoid and anthocyanin accumulation is highest on D2 (semi-open flower) stage and the cyanidins were the highest accumulated anthocyanidins. Additionally, other anthocyanidins were also present, where the dark purple color could be due to malvidins, peonidins, and petunidins. The differential expressions of major flavonoid and anthocyanin biosynthesis genes such as early biosynthesis genes (EBGs including, PAL, CHS, 5-O-(4-coumaroyl)-D-quinate 3′-monooxygenase (C3′H), FLS, F3H, HCT) and late biosynthetic genes (LBGs, including ANS, leucoanthocyanidin reductase (LAR), anthocyanidin reductase (ANR), DFR, anthocyanidin 3-O-glucoside 2'''-O-xylosyltransferase (UGT79B1), anthocyanidin 3-O-glucoside 6''-O-acyltransferase (3AT), anthocyanidin 3-O-glucosyltransferase (BZ1), and anthocyanidin 5,3-O-glucosyltransferase (GT1)) are controlling the differential anthocyanidin accumulation. We also discussed the potential roles of the genes enriched in plant hormone signal transduction pathway in changing the anthocyanidins accumulation. This combined omics approach elucidates the ABP related changes leading to the variations in anthocyanins’ accumulation in *H. syriacus* petals, thus proves to be an important resource for breeding Shigyoku or other varieties in this species in different shades.

## Material and method

### Plant material and growth condition

*Hibiscus syriacus* “Shigyoku” is the most popular flower in China. The research material was received from the Hunan Forest Botanical Garden. Plants were grown under normal conditions at the Chinese Academy of Agricultural Sciences (CAAS), 2021–22. The average temperature during the studied growing period was 29°C. Three distinct stages of flower development were selected, which are D1 (bud stage), D2 (early flowering stage which is also known as semi-open flower), and D3 (full bloom). For each stage, petals were collected in triplicate (single plants). The collected petals were stored at −80°C in liquid nitrogen and used for metabolic and transcriptomic analysis.

### Transcriptome analyses

#### RNA extraction, library preparation for transcriptome sequencing

Total RNA was isolated from nine samples using the TRlzol Reagent as per manufacturer’s protocol (Life Technologies, CA, United States). The RNA degradation and contamination were evaluated on agarose gel (1%). RNA purity was analyzed by using NanoPhotometer^®^ spectrophotometer (IMPLEN, CA, United States). RNA Assay Kit (Qubit^®^2.0 Fluorometer) was used to measure the RNA concentration. RNA integrity was determined by using a Bioanalyzer 2100 system (Nano 6000 Assay Kit Agilent Technologies, CA, United States). mRNA from total RNA was subjected to poly-T oligo attached magnetic beads for purification purpose. 1 µg RNA was taken for each sample and the libraries were prepared by using NEBNext^®^ UltraTM RNA Library Prep Kit Illumina^®^ (NEB, United States). The cDNA fragments of 250–300 base pair were selected.

Purification of the library fragments was done *via* using the AMPure XP system (Beckman Coulter, Beverly, United States). The random hexamer primer and M-MuLV Reverse Transcriptase (RNase H-) were used to synthesize the first strand of cDNA. Subsequently, second strand was prepared with the help of RNase H and DNA Polymerase I. Afterwards, the adaptor-ligated cDNAs with specific length were treated with 3 μl USER Enzyme (NEB, United States) for 15 min at 37°C and again for 5 min at 95°C. Then, PCR was carried out with the help of DNA polymerase and universal DNA primers. Finally, the PCR products were purified (AMPure XP system). Agilent Bioanalyzer 2100 system was used to assess the quality of the library. The cBot Cluster Generation System (TruSeq PE Cluster Kit v3-cBot-HS Illumia) was used for clustering the samples with index code as per manufacturer’s protocol. Afterward sequencing of libraries was performed on an Illumina Hiseq platform.

#### *De novo* transcriptome assembly and annotation

The Cassava 1.8 pipeline of the Illumina pipeline was used to filter the raw reads with paired ends. The low-quality reads and adapter reads were filtered. The *de novo* assembly of filtered reads was performed *via* adopting Trinity program 2.3.0 with a cut-off length >300 bp. Then, fastp v 0.19.3 (Ollion et al., 2013) software was used to filter the original data and the paired end reads, reads with adapters, and N content >10% eliminated. The low-quality paired-end (Q20>50%) reads were also removed, clean reads were obtained, and used for downstream analyses. Trinity (v 2.11.0) software (Grabherr et al., 2011) was used for transcriptome assembly (Kim et al., 2017). The transcript after Trinity assembly and de-redundancy was used as the reference sequence, and the clean reads of each sample were aligned to the reference sequence. For this, we used RSEM software (Li and Dewey, 2011), bowtie2 used in RSEM (Langmead and Salzberg, 2012). Finally, the appropriate transcripts were regrouped into gene clusters (unigenes) using Corset v 1.0.7 (Davidson and Oshlack, 2014).

#### Gene annotation, identification of DEGs

DIAMOND BLASTX program (Kanehisa et al., 2007) was used to compare the unigene sequences with KEGG, NR, Swiss-Prot, GO, COG/KOG, and TRembl databases., The unigenes’ amino acid sequences were predicted. Next, the HMMER software was implemented to compare them with Pfam database to obtain the annotation data of unigenes. To appraise and normalize the transcript expression level, RSEM tool (Li and Dewey, 2011) was adopted and then fragments per kilo base of transcript per million mapped fragments value (FPKM) were calculated. DESeq2 v1.22.2 (Love et al., 2014; Varet et al., 2016) was used for DEGs analysis. The *p-*value was normalized *via* implementing Benjamini & Hochberg method (multiple hypothesis testing) to attain the False Discovery Rate (FDR). To identify the differential genes, log_2_Fold Change ≥ 1 and the FDR value less than 0.05 were used as selection parameters.

### qRT-PCR analysis of anthocyanin related genes

We further studied the expression of the 15 anthocyanin-related genes in the petals on three stages i.e., D1, D2, and D3. The genes were selected based on their RNA-sequencing profile and relevance to their roles in the pathway. The primers for the selected genes were designed using Primer 3Plus software (Untergasser et al., 2007). First-strand cDNA synthesis kit (ThermoFisher Scientific, United States) was used. The reaction mixture preparation and PCR reactions were performed as reported earlier (Niu et al., 2017). The *Actin6* gene was used as a reference. The correlation was computed between the RNA-seq data and qRT-PCR data of the respective genes (Everaert et al., 2017).

### Metabolome analyses

#### Sample preparation for metabolomics

The petal samples were vacuum freeze-dried in a lyophilizer (Scientz-100F) and ground to powder with a grinder (MM 400, Retsch) at 30 Hz for 1.5 min. Afterwards, 100 mg powder sample was taken and mixed into 1.2 ml of 70% methanol extract. The solution was then vortexed for six times and placed in a refrigerator at 4°C overnight. Next morning, the samples were centrifuged at 12,000 rpm for 10 min and the supernatant was discarded. The samples were filtered through microporous membrane (0.22 μm) and transferred to the injection flask for UPLC-MS/MS analysis.

#### Conditions for chromatographic and mass spectrometry of analysis

Ultra-high-performance liquid chromatography (UHPLC) (SHIMADZU Nexera X2, and tandem mass spectrometry MS/MS) (Applied Biosystems 4500 QTRAP) were the basic system used for metabolite detection. The conditions for analysis are given in Table 1.

**TABLE 1:** UHPLC conditions for analysis.

UHPLC conditions	Specification
The chromatographic columns	Agilent SB-C18 1.8 μm, 2.1 mm* 100 mm
Mobile phase	Phase A: is ultrapure water (add 0.1% formic acid)
	Phase B: is acetonitrile (add 0.1% formic acid)
Gradient program	0 min 95:5 (v:v)
	9.0 min 5:95 (v:v)
	10.0 min 5:95 (v:v)
	11.0 min 95:5 (v:v)
	14.0 min 95:5 (v:v)
Flow velocity	0.35 ml/min
Temperature	40°C
Volume of Injection	4 μl

The effluent was coupled to an ESI-triple quadrupole-linear ion trap (QTRAP)-MS. Whereas, Table 2 shows the key components of mass spectrum conditions.

**TABLE 2:** Mass spectrometry analytical conditions.

Mass spectrometry condition	Components
Ion source	Turbo spray
Temperature of source	550°C
Ion spray voltage	5500 V
Source of ion	Gas I 50 psi
	Gas II 60 psi
	Curtain gas 25 psi
Collision gas	Medium

Each ion pair was scanned and identified using the optimal de-clustering potential (DP) and collision energy in triple quadrupole (QQQ) (Ma et al., 2021).

The qualitative evaluation of the secondary spectrum data was done by using a self-built database MWDB (Metware Biotechnology Co., Ltd. Wuhan, China). Both isotopic and duplicate signals comprising of following ions such as NH_4_^+^, K^+^, and Na^+^ and fragments (large molecular weight) were eliminated from analysis. The quantitative examination of metabolites was carried out using multiple reaction monitoring (MRM) analysis of QQQ-MS. Following the collection of metabolite data from several samples, the peak area of all metabolite mass spectra was integrated. Then, mass spectra of the same metabolites in different samples were combined and normalized. Differential metabolites were annotated and demonstrated by means of KEGG database.

#### Statistical analysis

R software was used for cluster analysis, as well as PCA following previously described methods (Ma et al., 2021). The differentially accumulated metabolites (DAMs) were selected based on the variable importance projection (VIP) and log2 fold change (log2FC) values. Where, the metabolites were significantly differentially accumulated if VIP ≥ 1 and log2 FC ≥ 1. The VIP values were taken from the Orthogonal Partial Least Squares Discriminant Analysis (OPLS-DA) results. The OPLS-DA was done using the R package in MetaboAnalystR. For this, the data were log-transformed and means were centered before OPLS-DA. The overfitting was avoided by conducting a permutation test (200 permutations) on the data.

## Results

### Comparative metabolome profile of H. syriacus “purple jade” petals

#### Overview of metabolome analysis

**FIGURE 1:**
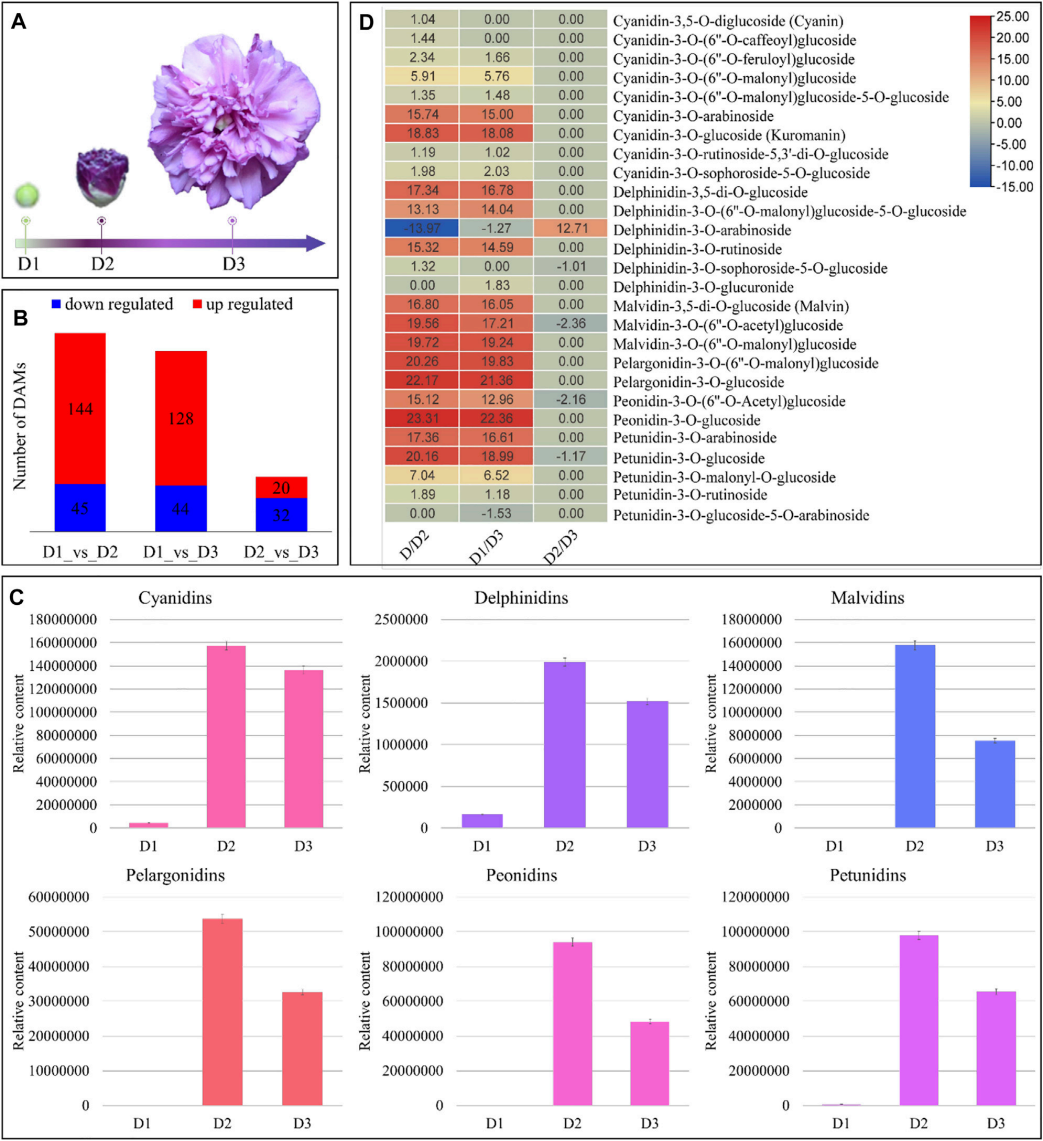
Metabolome analysis of *H. syriacus* petals on different days. **(A)** Flower samples on D1 to D3. **(B)** Summary of differential metabolite accumulation, **(C)** total contents of individual anthocyanidins on D1 to D3. The bars represent the mean of data from three HPLC-MS/MS runs, and **(D)** heatmap of anthocyanidins’ log2FC values between three stages’ comparisons. D1 (bud stage), D2 (early flowering stage/semi-open flower), and D3 (full bloom) represent flower development stages. The colors of the bars in “c” represent respective pigment coloration. The error bars represent standard deviation.

The HPLC-MS/MS-based metabolome profiling of the fresh *H. syriacus* purple jade petals (Figure 1A) revealed the differential accumulation of 189, 172, and 52 metabolites; in total, we detected 301 metabolites (Figure 1B). The differentially accumulated metabolites (DAMs) were those whose FC was ≥ 2 or ≤ 0.5 between the comparative groups. The principal component analysis (PCA) grouped the metabolites in three separate clusters indicating the reliability of sampling (Supplementary Figure S1). The DAMs were enriched in metabolic pathways, flavonoid biosynthesis (and related pathways), biosynthesis of secondary metabolites, and anthocyanin biosynthesis (Supplementary Figure S2).

#### *H. syriacus* petals undergo major changes in flavonoid and anthocyanin accumulation from D1 to D3

Since the major color contributing pigments in *H. syriacus* are the anthocyanins, therefore, we focused on the DAMs that were enriched in both the flavonoid biosynthesis and ABP. The highest flavonoid contents were present in D1 followed by D2, and D3. The higher content in D1 was due to gallocatechin, otherwise, the flavonoid accumulation trend was D2>D3>D1 (Supplementary Table S1). The HPLC-MS/MS analyses-based quantification showed that almost all anthocyanidins had lowest content (or were not present) on D1, increased significantly (maximum accumulation) in D2, and finally their contents decreased on D3 (relative to D2) (Figure 1C). Overall, 25, 24, and five anthocyanins were differentially accumulated between D1vsD2, D1vsD3, and D2vsD3, respectively (Figure 1D). Major anthocyanin groups detected in metabolome analysis were cyanidins, delphinidins, malvidins, pelargonidins, peonidins, and petunidins. On D1, only cyanidins, delphinidins, and petunidins were accumulated; cyanidins being the highest followed by petunidins, and delphinidins. The cyanidins content increased up to 18.83 log2FC in D2 as compared to D1. Similarly, the delphinidin, malvidin, pelargonidin, peonidin, and petunidin content increased by 17.34, 19.72, 22.17, 23.31, and 20.16 log2FC, respectively, from D1 to D2. These results indicate that almost all anthocyanins were produced during the transition from D1 to D2. However, the darker coloration on D2 could be due to the accumulation of malvidins, pelargonidins, and peonidins. Similarly, these pigments showed higher accumulation in D3 as compared to D1. From D2 to D3, we observed a reduced accumulation of cyanidins, delphinidins, malvidins, pelargonidins, peonidins, and petunidins (Figure 1D). Whereas we noted increased accumulation of cyanidin-3-(6″-malonyl)glucoside-5-glucoside, cyanidin-3-sophoroside-5-glucoside, delphinidin-3-(6″-malonyl)glucoside-5-glucoside, delphinidin-3-arabinoside, delphinidin-3-glucuronide, and petunidin-3-o-glucoside-5-o-arabinoside in D3 as compared to D2. Nevertheless, the cumulative content of the anthocyanidins was highest than the other pigments on D3.

Overall, the metabolome profile of *H. syriacus* flowers show that flavonoid and anthocyanin accumulation is highest on D2, which then decreases on D3. Cyanidins (the highest accumulated anthocyanidin), delphinidins, malvidins, pelargonidins, petunidins, and peonidins are major color contributing anthocyanidins. The higher concentrations of these anthocyanidins (malvidins, peonidins, and petunidins) impart darker purple color (on D2 stage), which then turns purple (lavender) on D3 owing to reduction in anthocyanidin content (Figure 1).

### Transcriptome profiling of H. syriacus petals on different days

#### Overview of transcriptome sequencing

**FIGURE 2:**
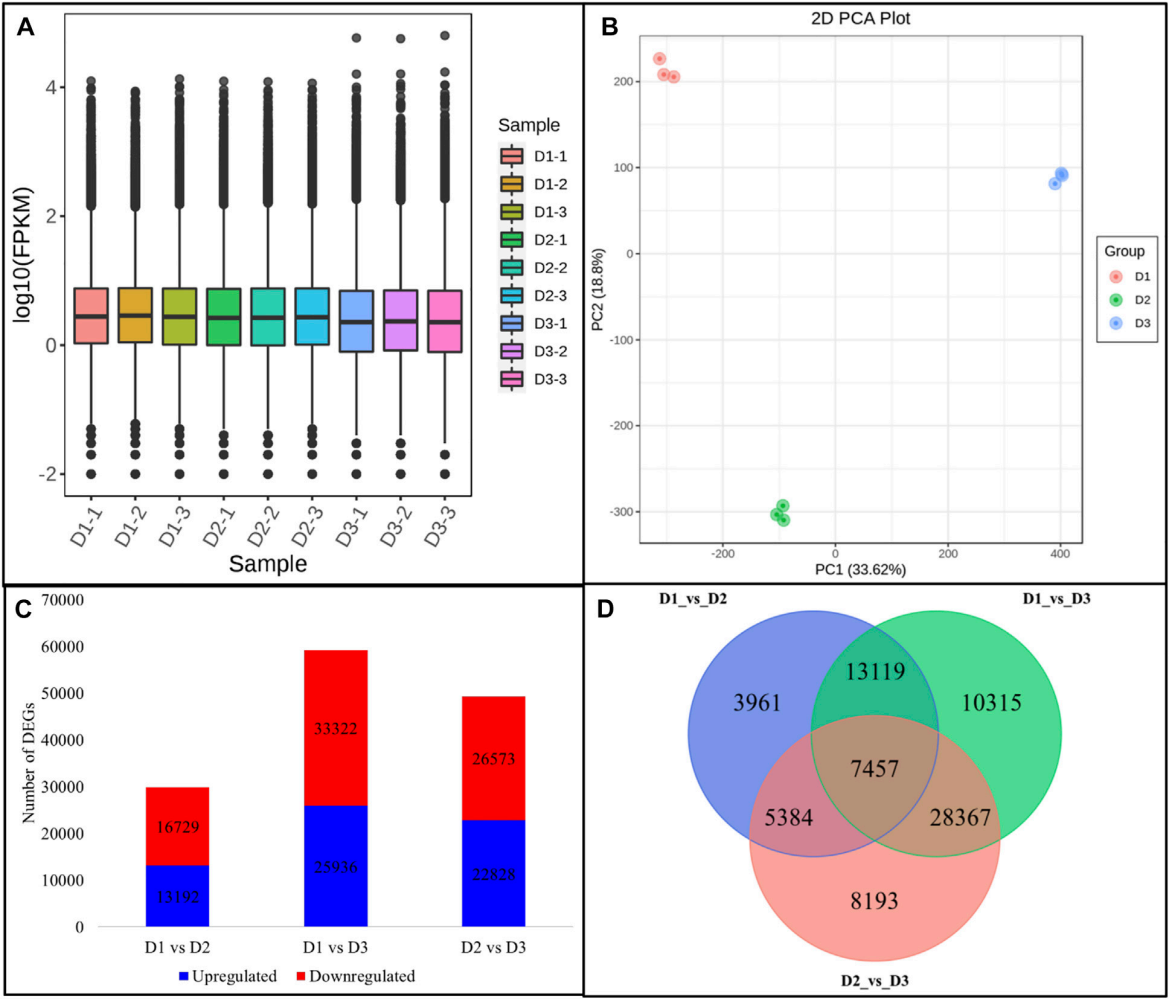
Overview of *H. syriacus* petal transcriptome. **(A)** Distribution of the gene expression in petals on three different developmental stages. **(B)** Principal component analysis of the expressed genes. **(C)** Statistics of the differentially expressed genes between petals harvested on different developmental stages. **(D)** Venn diagram representing a common and specific number of DEGs between the three comparisons. Where, D1 (bud stage), D2 (early flowering stage), and D3 (full bloom) represent flower development stages.

Global gene expression profiling of the *H. syriacus* petals was done by transcriptome sequencing. The nine cDNA libraries produced 58.97 Gb clean reads (an average of 43.68 million clean reads per library). 73.81% of the clean reads could be mapped onto the reference sequence (the transcripts after Trinity assembly and de-redundancy were used as the reference sequence). The error rate and GC contents were 0.03 and 44.5%, respectively (Supplementary Table S2). The sequencing produced 313,323 transcripts and 303,832 unigenes; all the unigenes could be annotated (Supplementary Figure S3). Overall FPKM values on D1 and D2 were higher than on D3 (Figure 2A). The PCA analysis grouped the respective replicates of each flowering stage together suggesting the reliability of the sampling (Figure 2B). There were 29,921, 59,258, and 49,401 DEGs in D1vsD2, D1vsD3, and D2vsD3, respectively (Figure 2C); 7,457 of which were common between the three comparisons (Figure 2D).

We looked for the top-10 DEGs that were upregulated in D2 as compared to D1 since anthocyanin accumulation was significantly higher than D1. Hsp70 kDa protein 2-like was the most upregulated gene in D2 followed by ubiquitin carboxyl-terminal hydrolase 36, Ubiquitin-protein ligase 24, xylan 1,4-beta-xylosidase, bidirectional sugar transporter SWEET10-like, minor food allergen Cap a 2, dihydroceramide fatty acyl 2-hydroxylase, peroxidase 21, and a glutamate receptor 2.3-like. Whereas the top-10 genes that were upregulated in D3 as compared to D2 are 1-deoxy-D-xylulose-5-phosphate synthase, MYB308-like, multidrug resistance protein (MATE family), gibberellin 2-oxidase (Ga2ox), two DNA-directed RNA polymerase II subunit RPB1, syntaxin 1B/2/3, SAUR family protein, and lachrymatory-factor synthase-like (LFS-like). These observations indicate that from D1 to D2 the processes such as ubiquitination, changes in cell wall, and sugar transport are involved. These process could be relevant to the developmental mechanisms and may also be related to ABP. On the contrary, the upregulation (or exclusive expression) of MYB, Ga2ox, SAUR, and LFS-like indicates that hormone signaling (GA signaling in particular), anthocyanin transport, and stability are highly regulated from D2 to D3 (Supplementary Table S3).

KEGG pathway enrichment showed that DEGs were significantly enriched in metabolic pathways, biosynthesis of secondary metabolites, flavonoid biosynthesis, starch and sucrose metabolism, and signaling-related pathways (plant hormone signal transduction and MAPK signaling pathway) (Supplementary Figure S4).

#### Expression changes in anthocyanin biosynthesis genes is consistent with the respective metabolite accumulation in *H. syriacus* petals

**FIGURE 3:**
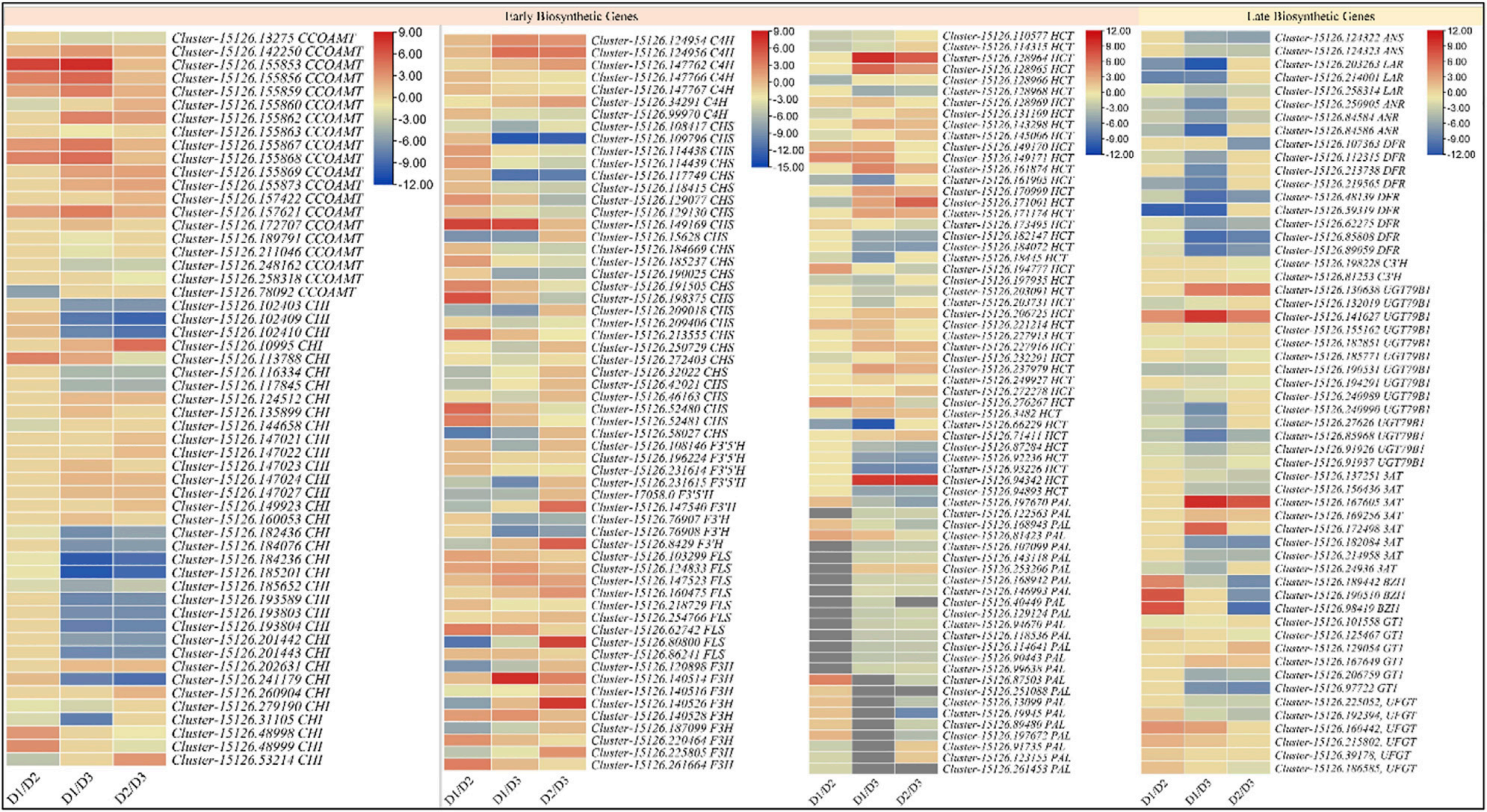
Heatmaps (log2 foldchange) of the anthocyanin biosynthesis genes. D1 (bud stage), D2 (early flowering stage), and D3 (full bloom) represent flower development stages.

The transcriptome analyses showed that 178 DEGs were significantly enriched in the flavonoid biosynthesis pathway; 98, 141, and 129 DEGs in D1vsD2, D1vsD3, and D2vsD3, respectively. For the ABP, we found 16, 26, and 21 DEGs enriched in D1vsD2, D1vsD3, and D2vsD3, respectively. Among the EBGs, 25 DEGs annotated as PAL were differentially regulated in the studied tissues. Most PAL transcripts showed upregulation in D2 as compared to D1, and downregulation in D3 as compared to D1 and D2. This is consistent with the anthocyanidins accumulation patterns in the respective stages. Trans-cinnamate 4-monooxygenase (C4H), CHSs, CHIs, and F3Hs showed variable expression trends in the three developmental stages. All the F3′5′H transcripts were all downregulated in D2 and D3 as compared to D1. However, it is to be noted that only two transcripts (though different) were differentially expressed in D1vsD2 and D2vsD3 suggesting that a larger number of F3′5′Hs are highly expressed in D1 as compared to D2 or D3. Overall, the upregulation in D2 (as compared to D1) and downregulation in D3 (as compared to D2) of eight PALs, six CHS, a C3′H, two FLS, two F3H’s, and three HCTs is consistent with the anthocyanidins’ accumulation (Figure 3 and Supplementary Table S4).

Among the LBGs, two ANSs (*Cluster-15126.124322*, and *Cluster-15126.124323*) were slightly upregulated in D2 as compared to D1 but their expression significantly reduced in D3. The LAR transcripts had higher expressions on D1, which then reduced on D2. Most LARs didn’t express in D3. The ANRs showed downregulation in D2 as compared to D1 but were not expressed in D3. These expression changes indicate that leucoanthocyanins are reduced to (+)-afzelechin, (+)-catechin, and (+)-gallocatechin in D1 but in D2 the ANS allow the biosynthesis of anthocyanins/anthocyanidins. Furthermore, a DFR (*Cluster-15126.107363*) was exclusively expressed in D2, while eight others were highly expressed in D1. Additionally, three DFRs were downregulated in D3. The expression patterns indicate that DFRs are activated on D1 and their expression continue to decrease with time. Furthermore, we noted that C3′H, UGT79B1, 3AT, BZ1, and GT1 were also differentially regulated between the three stages. The C3′Hs were expressed only in D2 and D3, where the expressions were higher on D2. These are consistent with higher dihydroquercetin biosynthesis in D2 as compared to D3. Two UGT79B1’s (*Cluster-15126.141627* and *Cluster-15126.130638*) showed strong expressions on D3, whereas their expression on D1 and D2 were fractional. These expressions support the results that D3 had reduced contents of 3-O-glucosides in D3 as compared to D2. This is further supported by the most important observation that two BZ1s (*Cluster-15126.190510* and *Cluster-15126.98419*) were exclusively expressed in D2 and a third (*Cluster-15126.189442*) was highly upregulated in D2 as compared to D1. Most 3ATs’ expressions increased from D1 to D2 and then significantly decreased on D3, which is consistent with the accumulation pattern of cyanidin-3-o-(6″-o-caffeoyl)glucoside. The reducing 3-o-glucoside contents from D2 to D3 are also consistent with the 3AT expression trends. The expression of two GT1s (*Cluster-15126.125467* and *Cluster-15126.97722,* particularly, the second transcript) is highly correlated with the cyanidin-3,5-o-diglucoside. The GT1s expressions were variable i.e., some transcripts had a higher expression on D1 as compared to D2 and D3, but the others showed contrasting expressions. This is because this enzyme regulates two successive steps i.e., the conversion of cyanidin to cyanidin-5-o-glucoside and its further conversion to cyanidin-3,5-o-diglucoside. Since we noted the higher accumulation of glycosylated anthocyanins in D2 as compared to D1, therefore we explored if there were any flavonol 3-O-glucosyltransferases (UFGTs). Interestingly, five UFGTs showed upregulation in D2 as compared to D1, whereas one showed contrasting expression i.e., higher in D1 as compared to D2. On the other hand, only two of six were upregulated in D3 as compared to D1. These expression changes are consistent with the metabolite results (Figure 3 and Supplementary Table S4).

Seemingly, the expression trends of LARs, ANSs, DFRs, UGT79B1, C3′Hs, 3ATs, and BZ1s are highly consistent with that of the respective metabolite accumulation. Additionally, the expressions of the UFGTs are relatable to the higher anthocyanin accumulation in D2. More specifically, the UFGTs are responsible for higher glycosylated anthocyanins in D2 and D3 as compared to D1.

#### Expression changes in plant hormone signal transduction pathway genes

Hormone signaling has been strongly associated with the regulation of the expression of ABP-related genes. Furthermore, hormone signaling also affects the expression of the ABP activators/repressors. Considering these important roles and the fact that DEGs were significantly enriched in the plant hormone signal transduction pathway, we specifically looked into this pathway. We noted the differential expression of 1184, 1587, and 1585 genes in D1 vs. D2, D1 vs. D3, and D2 vs. D3, respectively; overall 2469 DEGs were enriched in this pathway. The observation that a large number of DEGs were enriched in this pathway is interesting and implies large-scale changes in the hormone signaling pathway, which in turn suggests important roles in the ABP as well as growth and development in the *H. syriacus* flower. Since we noticed that anthocyanin biosynthesis was highly increased on D2 and then reduced on D3, therefore, we specially looked for plant-hormone signaling-related DEGs that show corresponding expression trends (Supplementary Table S4).

JAZ’s (Jasmonate ZIM domain-containing protein) attachment with COI1 (coronatine-insensitive protein 1) allows it to detach from the MBW complex, which is then transcriptionally activated (Garrido-Bigotes et al., 2020). Of 20 differentially expressed JAZ transcripts, 75% (15) showed reduced expression in D2 as compared to D1, which indicates JAZ’s are being degraded in D2. Of these 20, only nine were differentially expressed between D2 and D3, whereas five were upregulated in D3. Only two COI1 transcripts were differentially expressed in D1 vs. D2; *Cluster-15126.193655* was highly expressed in D2 as compared to D1. Interestingly, 12 COI1s were differentially expressed between D2 and D3, where all of these were downregulated in D3. These expression trends indicate that JAZ’s are degraded in D2, which leads to higher anthocyanin biosynthesis, whereas the contrasting expression leads to lower anthocyanin accumulation in D3. From this expression pattern, we can understand that JAZ degradation leads to the transcriptional activation of MBW complex in *H. syriacus* flower resulted in color changes (Supplementary Table S4).

Regarding auxin signaling, we noted that 36 of the 46 TIR1s (transport inhibitor response 1) showed decreased expression in D2 and D3 as compared to D1. This expression trend is contrasting to the ABP genes’ expressions. Whereas, the upregulation of a 108 ARF (auxin response factor) transcripts in D1 could be a signal to repress ABP genes in D1 and not in D2. This correlates with the lower expression of 50 IAA (auxin-responsive protein IAA) transcripts in D1 as compared to D2. Thus, IAA’s higher expression in D2 suggests that they bind with ARFs to remove their repressive action on ABP genes (Supplementary Table S4).

A total of 44 DELLAs were upregulated while 36 were downregulated in D2 as compared to D1. The higher expression of a larger number of GID1 and GID2 transcripts in D1 and reduced expression in D2 indicates that in D1 GID1’s degrades a larger number of DELLAs but not in D2. The degradation of DELLAs in D1 could be a negative regulator of the anthocyanin biosynthetic genes. Similarly, 66 DELLAs were downregulated in D3 as compared to D2. The higher expression of the other DELLA transcripts in D1 and the contrasting expression of GIDs is understandable since we also detected the anthocyanin accumulation in D1 (Supplementary Table S4).

#### Changes in the expression of transcription factors and MBW complex-related DEGs

The transcription factor (TF) annotation and classification indicated the differential expression of 82 (2,648 DEGs), 87 (4,164 DEGs), and 85 (3,280 DEGs) families in D1vsD2, D1vsD3, and D2vsD3, respectively; 646 were commonly regulated between the three stages. The top-10 upregulated TFs in D2 as compared to D1 were AP2/ERF, bHLH, MYB-related, WRKY, PHD, MYB, and MADS-MIKC. On the contrary, the top-10 downregulated TFs in D3 as compared to D2 were classified as MYB, bHLH, OFP, AP2/ERF-ERF, and C2C2-GATA. The differential expression of the MYB/MYB-related and bHLH TFs is interesting since both of these are the MBW complex components, which are known as the ABP genes’ regulators in different plant species. Thus, we checked the other transcripts annotated as the MBW complex components i.e., MYB, WDR, and bHLH; WDR were not differentially expressed between the studied stages. In D1vsD2, 84 MYB TFs were differentially expressed; 52 were upregulated, while 32 were downregulated in D2 as compared to D1. Of these, one MYB TF (MYB114, *Cluster-15126.103342*) and three MYB-related TFs (TRB1, *Cluster-15126.64686*; MYB92; *Cluster-12612.0*; and MYBS3, *Cluster-15126.251292*) showed exclusive expression in D2. Only MYBS3 showed higher expression in D3 as compared to D2 but the rest didn’t express in D3. These MYB TFs can be positive regulators of anthocyanin biosynthesis. Additionally, the upregulation of a higher number of 109 MYB TFs (as compared to 87 downregulated MYB TFs) in D2 as compared to D1 clearly indicates that more MYB TFs are active in D2 as compared to D1 and thus influencing transcriptional regulation of anthocyanin biosynthetic genes. In case of D2vsD3, 120 MYB TFs were differentially expressed; 59 were downregulated whereas 61 were upregulated in D3. The specific MYB TFs in D3 were MYB91, 106, 1, 14, 63, 73, 88, DIVARICATA, ODO1, and SRM1 (Supplementary Table S5).

Three hundred and seventeen bHLH TFs were differentially expressed between the replicates; 67 and 101 were up- and downregulated in D2 as compared to D1, respectively. Top five bHLH TF in D2 were bHLH092, bHLH072, bHLHAs. Whereas in D3, we observed the upregulation of bHLH9, bHLH120, and bHLH72. Four of these (bHLH072, *Cluster-15126.161369*; bHLH072, *Cluster-15126.158188*, bHLHA, *Cluster-15126.142146*, and *Cluster-15126.281970*) were expressed in D2 and D3. Since the anthocyanin content was highest on D2 and then decreased on D3, therefore, we specifically looked for the bHLH TFs that showed similar expression trends; 46 bHLH TFs showed higher expression on D2 as compared to D1, which then decreased on D3 (Supplementary Table S5).

Overall, these expression analyses indicate that in *H. syriacus* a large number of MBW complex related transcripts (MYB and bHLH) takes part in anthocyanin biosynthesis.

### qRT-PCR analysis validates transcriptome and metabolome analyses

**FIGURE 4:**
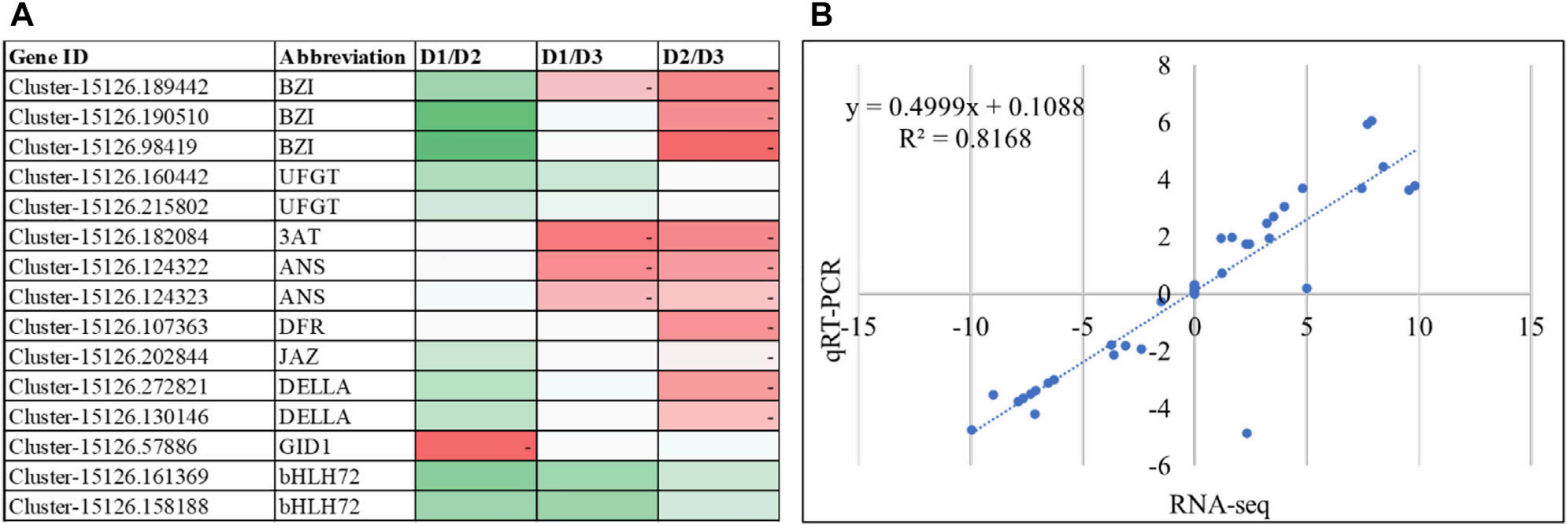
**(A)** qRT-PCR expression of the anthocyanin related genes. The heatmaps show change in expression from one stage to the other. The color of the boxes represents lower (green) to higher (red) relative change values. **(B)** Correlation between qRT-PCR data and RNA-seq (FPKM values) of the selected genes.

The qRT-PCR analysis of selected genes related to anthocyanin biosynthesis revealed similar expression patterns as RNA-seq data (Figure 4A). The correlation between the FPKM values and qRT-PCR expression was 0.8168 signifying that the latter is consistent with the RNA-seq results (Figure 4B). These observations reaffirm the transcriptome and metabolome analysis results that anthocyanin content varies in the three stages of *H. syriacus* petals and is responsible for the change in color.

### Co-joint analyses of RNA-sequencing and metabolite profiling confirm accumulation patterns of anthocyanins

The relationship between the differentially expressed transcripts and accumulated metabolites between the studied stages of *H. syriacus* petals was further explored through a co-joint analyies. Most importantly, we first looked for the KEGG pathways to which the DEGs and DAMs were jointly enriched. Six KEGG pathways i.e., flavonoid biosynthesis, anthocyanin biosynthesis, isoflavonoid biosynthesis, flavone and flavonol biosynthesis, metabolic pathways, and biosynthesis of secondary metabolites were the significantly enriched pathway (Supplementary Figure S5). A detailed look into the flavonoid biosynthesis pathway and ABP confirmed the individual results of RNA sequencing and metabolite profiling. More specifically, we observed that the upregulation of BZ1 transcripts lead to the increased accumulation of pelargonidin 3-glucoside, pelargonidin 3-malonyl-glucoside, cyanidin 3-malonyl-glucoside, peonidin 3-glucoside, cyanidin 3-glucoside, cyanidin 3,5-glucoside, petunidin 3-glucoside, delphinidin 3-rutinoside, and delphinidin 3,5-diglucoside (Supplementary Figure S6).

## Discussion

### Petal color transition is attributed to the changes in the accumulation of the major anthocyanins

We adapted a combined metabolome and transcriptome approach and explored the metabolic and transcriptomic changes in petal from D1 to D3 (Figure 1A). Recent research on *H. syriacus* flowers has revealed presence of as many as 40 different anthocyanin components; particularly cyanidin, delphinidin, procyanidin, peonidin, pelargonidin, petunidin, and malvidin have been detected (Zhang et al., 2022). Our results that all the major anthocyanins were present in *H. syriacus* petals, are consistent with previous findings (Figure 1). The detection of cyanidins, petunidins, and delphinidins on D1 indicates that ABP was already active before flower opening, which is a common phenomenon in most of the flowering plants (Justesen et al., 1997). The purple coloration of the *H. syriacus* petals in our study could be associated with the increased accumulation of these anthocyanins. However, the significantly higher accumulation of the glycosylated forms of cyanidin, delphinidin, malvidin, petunidin, pelargonidin, and peonidin seems to be the major color contributor in D2 (Figure 1D). The glycosylated forms are produced when sugar moieties are attached to the unstable anthocyanidin aglycones (Cheng et al., 2014). This glycosylation stabilizes anthocyanins and also serves as signal for their transport to vacuoles that allows them to function as pigments (Mathews et al., 2003).

### Expression changes in the key structural genes causes the variations in the accumulation pattern of the anthocyanins and the resultant color change

**FIGURE 5:**
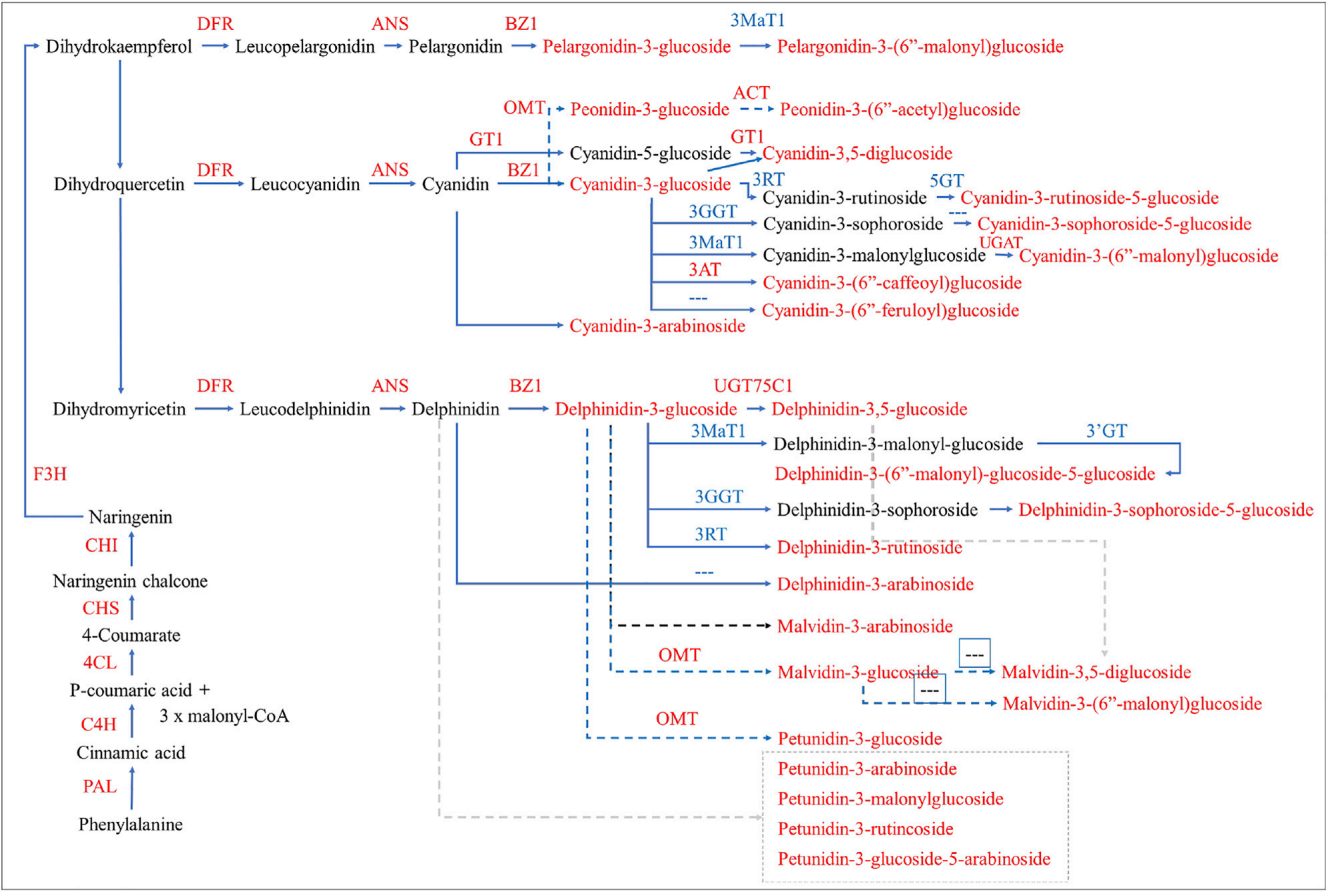
Differential regulation of anthocyanin biosynthesis in *H. syriacus* petals. The genes and metabolites that were differentially expressed and accumulated, respectively, are given in red text. The dotted arrows/lines indicate that the genes are not found or not known. The pathway was reconstructed by following the layout and nomenclature available on https://www.genome.jp/kegg/kegg2.html. The gene name abbreviations correspond to the full names given in Supplementary Table S4.

The EBGs and LBGs control the major steps in anthocyanin biosynthesis (Liu et al., 2018). This means that the flavonoid biosynthesis pathway leads to the production of anthocyanins (LaFountain and Yuan, 2021). As a first step, the phenylalanine is converted into cinnamate by the action of PAL. The expressions changes in PAL transcrips in D2 (as compared to D1) indicate that the differential anthocyanin biosynthesis starts way upstream the pathway, this is consistent with the relationship of PAL with the accumulation of different anthocyanins in tea (*Camellia sinensis* L.) (Chen et al., 2021). Further down in the pathway, the reduced expression of C3′Hs in D3 as compared to D2 indicates that reduced biosynthesis of the active dihydroflavonol intermediate (dihydroquercetin) is one of the causes of reduced anthocyanin accumulation in D3 (Gang et al., 2002). On the other hand, the varied expressions of EBGs i.e., C4H, CHS, CHI, and F3H confirm our above statement that the anthocyanin biosynthesis had already started in D1, however, they are not the major cause in the anthocyanin accumulation pattern. Nevertheless, the consistent upregulation of PAL, CHS, C3′H, FLS, F3H, and HCT transcripts with the anthocyanin accumulation patterns proposes their important roles as reported in other flowering plants i.e., *Paeonia lactiflora* (Zhao et al., 2012), *Hibiscus cannabinus* L. (Lyu et al., 2020), and other ornamental plants [reviewed in (Zhao and Tao, 2015)]. Furthermore, the reducing expressions of LARs in D2 indicate that the pathway is not moving in the direction of the biosynthesis of flavan-3-ols i.e., (+)-afzelechin, (+)-catechin, and (+)-gallocatechin (Tanner et al., 2003). Instead, the ANS upregulation in D2 causes higher anthocyanin biosynthesis and their downregulation in D3 is responsible for the opposite i.e., reduced anthocyanin biosynthesis (Figure 5).

The succeeding expression changes in DFR further strengthen our statement that the anthocyanin biosynthesis was already active in D1. Also, the downregulation of three DFRs in D3 can be associated with the reduced anthocyanin accumulation (Figure 1C) (Li et al., 2017) because, a purple sweet potato DFR has been characterized for a similar role in anthocyanin biosynthesis (Wang et al., 2013). The increased biosynthesis of 3-O-glucosides of pelargonidin, cyanidin, and delphinidin in D2 and then their decrease in D3 changes can be explained by the changes in BZ1s expressions. BZ1 catalyzes the transfer of the glucosyl moiety from UDP-glucose to the 3-hydroxyl group of the anthocyanidins as reported in *Iris hollandica* (Yoshihara et al., 2005). Furthermore, the reducing 3-O-glucosides and increasing accumulation of cyanidin-3-O-(6″-O-caffeoyl)glucoside in D3 as compared to D2 is consistent with the 3AT transcripts’ expression (Yonekura-Sakakibara et al., 2000). Another explanation of the higher accumulation of the glycosylated anthocyanins in D2 and then their reduction in D3 is the up and downregulation of UFGTs in both stages, respectively. These results are consistent with the UFGT’s functions in most of the flowering plants e.g., *Freesia hybrida* (Sun et al., 2016) (Figure 5).

Hence, our combined metabolome and transcriptome analyses indicate that the ABP was active in D1 as evident from the expression of EBGs and LBGs. The higher anthocyanidins accumulation in D2 as compared to D1 and reduced accumulation in D3 as compared to D2 is due to the changes in the expressions of EBGs and LBGs. Specifically, C3′Hs, LARs, ANSs, DFRs, UGT79B1s, BZ1s, and 3ATs seems to be controlling the major steps in the anthocyanin accumulation differences from D2 to D3 (Figure 5).

### The expressions of the MBW complex’s components are consistent with the of the changes in anthocyanin accumulation

Since *H. syriacus* is a dicot and the transcriptome sequencing results showed that both EBGs and LBGs take part in the differential anthocyanin accumulation and resultant petal coloration (as explained in the above sections). Therefore, it is necessary to understand the changes in the expressions of the MBW complex components. The upregulation of a very large number of MYB TFs in D2 is an indication of the active MBW complex. Similarly, the differential expressions of the MYB TFs in D1 further justify our proposition that the ABP was already active in D1. Likewise, the observations that MYB/MYB-related and bHLH TFs were among the top-10 highly upregulated DEGs in D2 and highly downregulated in D3 corresponds to the anthocyanin profiles on these stages. These results imply that MBW complex components are active in D1, however, their higher expressions in D2 possibly increasingly regulate the ABP genes (EBGs and/or LBGs), that lead the higher anthocyanin accumulation (Liu et al., 2018; LaFountain and Yuan, 2021; Yan et al., 2021). Contrastingly, the downregulation of MYBs and bHLHs in D3 can be linked with the reduced anthocyanins (Liu et al., 2018; LaFountain and Yuan, 2021; Yan et al., 2021). Thus, the metabolome data supported with the transcript expressions of ABP genes and MBW complex components indicates the mechanism of *H. syriacus* petal coloration (Yan et al., 2021). Most interesting observation was the exclusively expressed MYBs in D2. Particularly MYB114s activation can be associated with expression of many LBGs and resultant higher anthocyanin biosynthesis in D2. In apple and Arabidopsis, the MYB114 has been characterized for its role in the regulation of anthocyanins (Gonzalez et al., 2008; Jiang et al., 2021). The MYB114s expression has been shown to be bHLH dependent. Thus, it is possible that it interacted with the four exclusively expressed bHLHs (*Cluster-15126.158188, Cluster-15126.142146, Cluster-15126.161369*, and *Cluster-15126.281970*). The specific interaction of these TFs with anthocyanins needs further specific characterization experiments.

### Plant-hormone signaling pathway’s role in *H. syriacus* petal coloration

Hormone signaling pathway in plants have been studied in multiple species to understand the role of various hormone signals in the transcriptional activation/repressions of MBW complex and/or ABP genes (LaFountain and Yuan, 2021). Among all hormones, the effect of JA is majorly studied and it has been shown that the application of MeJA activates anthocyanin biosynthesis. This is because the JAZ proteins bind directly with the MYB and bHLH TFs (of the MBW complex) (Qi et al., 2011). When COI is expressed, it attaches to JAZ (through SCF complex) and degrades it (Pauwels and Goossens, 2011). Thus, if JAZ’s are expressed highly in a tissue, it is reasonable that anthocyanin biosynthesis would be low because the MBW complex is occupied. The results that 75% of the JAZ transcripts had higher expression in D1, which then reduced in D2 depicts that JAZ’s are being degraded in D2, while they are again upregulated in D3. These results are further supported with the expression trends of COI. Thus, the significant upregulation of anthocyanins on D2 stage of *H. syriacus* petals clearly correlates with the anthocyanin accumulation. This mechanism has been reported in *Fragaria vesca* L. x *F. ananassa* (Garrido-Bigotes et al., 2020). Other than JA signals, our results suggest that auxin signals can be another reason for the anthocyanin biosynthesis and resulting petal color. IAA121 and ARF13 when bound, release MBW complex from ARF13, which transcriptionally activates anthocyanin biosynthesis. When IAA121 is bound with TIR1, the ARF13 destabilizes the MBW complex as well as represses anthocyanin biosynthesis genes as studied in apple (Wang et al., 2018). The decreased expression of TIR1 transcripts is relatable to the increased expression of ABP genes in D2 as compared to D1. Similarly, the higher expression of a large number of ARFs (108 transcripts) in D1 suggests their repressive action on ABP genes (Wang et al., 2020). This is further supported by the reduced expressions of 50 IAA transcripts in D1 as compared to D2. Therefore, we can propose that similar to apple, the *H. syriacus* IAA’s bind with ARFs and remove their repressive action on ABP genes. Finally, the expression patterns of the DELLAs in the studied stages of *H. syriacus* petal indicates that GA signaling is also at play to change the anthocyanin biosynthesis and the resulting color (Qi et al., 2014; Xie et al., 2016). Nevertheless, these expression patterns of the JA, auxin, and GA signaling genes put forward a basic understanding of the hormonal control of the petal color formation. It gives us clues and provide us a list of multiple hormone-signaling associated genes and TFs that should be characterized and manipulated for desired color formation in *H. syriacus.*

## Conclusion

*Hibiscus syriacus* L. petal color transition from D1 to D3 was studied through metabolome profiling, transcriptome sequencing, and qRT-PCR analysis. The bluish-purple coloration of the petals on D2 appeared to be associated with significantly higher accumulation of anthocyanidins i.e., cyanidin, delphinidins, malvidins, pelargonidins, petunidins, and peonidins. The purple (lavender) petal color on D3 was due to reduced contents of these anthocyanidins. The transcriptome analysis showed that both EBGs and LBGs were differentially expressed. Based on the transcriptome sequencing results, the major genes that contributed to the changes in petal color were C3′Hs, LARs, ANSs, DFRs, UGT79B1s, BZ1s, and 3ATs. The transcriptomic signatures that were associated with the expression of these genes included the MBW complex components, particularly MYB and bHLH TFs. Furthermore, JA, auxin, and GA signaling related genes showed expressions that suggest the roles of respective hormones in affecting the MBW complex, ABP genes, and the resulting anthocyanin accumulation.

## Data Availability

The original contributions presented in the study are included in the article/Supplementary Material, further inquiries can be directed to the corresponding authors. The datasets for this study were deposited to the NCBI with the BioProject accession ID as “PRJNA851843”.
