# Supplementary figures and images for: An integrated metabolome and transcriptome analysis of the Hibiscus syriacus L. petals reveal the molecular mechanisms of anthocyanin accumulation
Source: Front Genet. 2022 Sep 5;13:995748. doi: 10.3389/fgene.2022.995748 (PMC9483124; doi:10.3389/fgene.2022.995748)

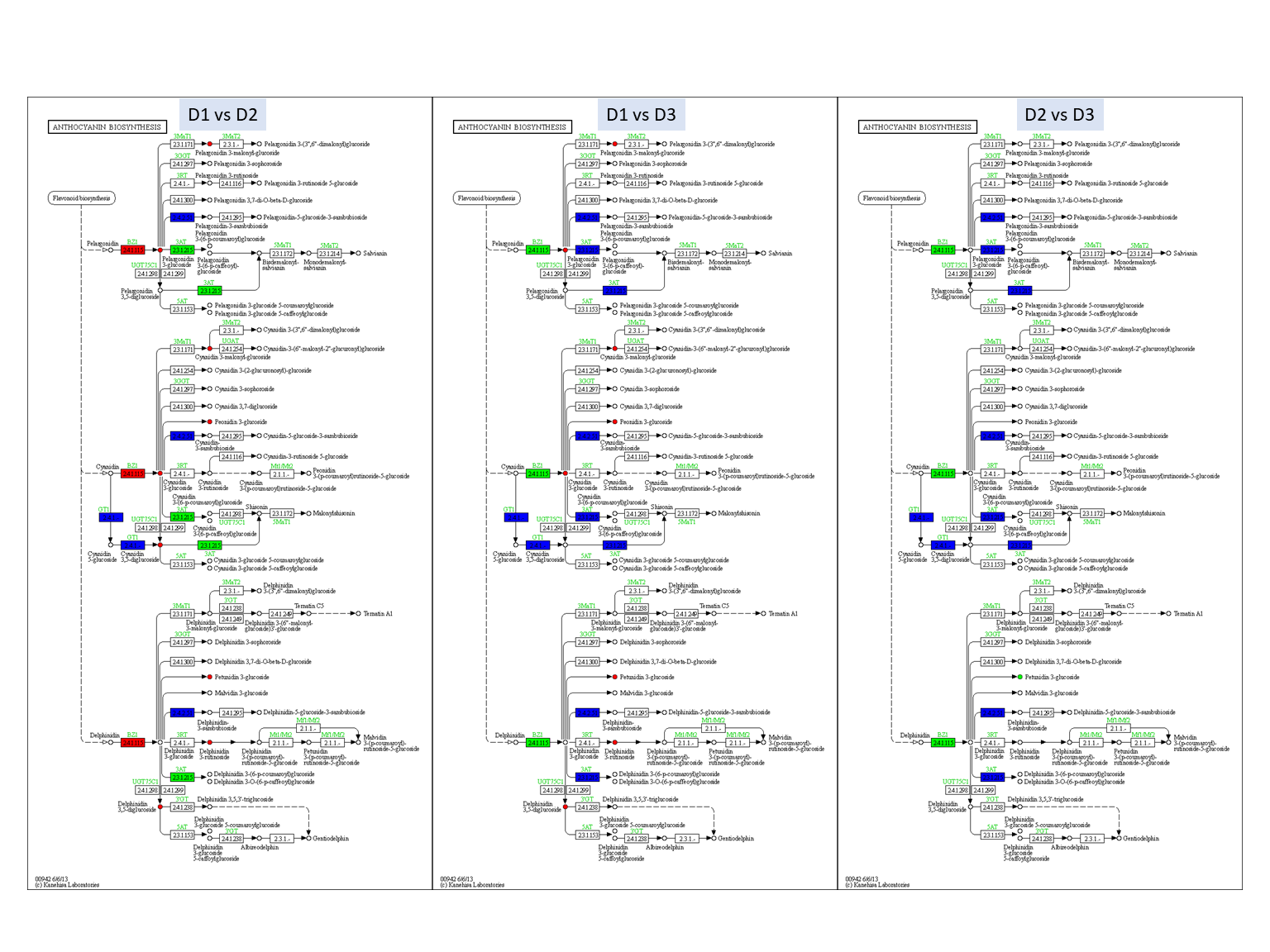

Supplement: Supplementary file 1 [file Image6.TIF]

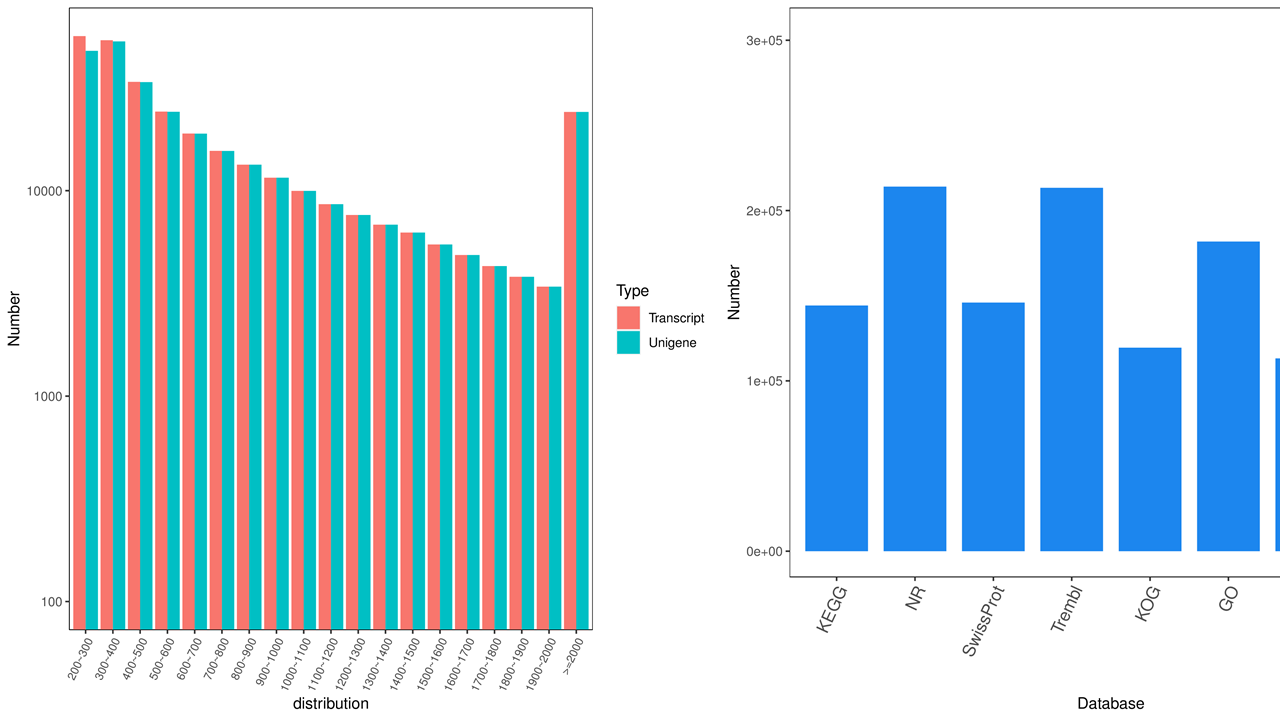

Supplement: Supplementary file 2 [file Image3.TIF]

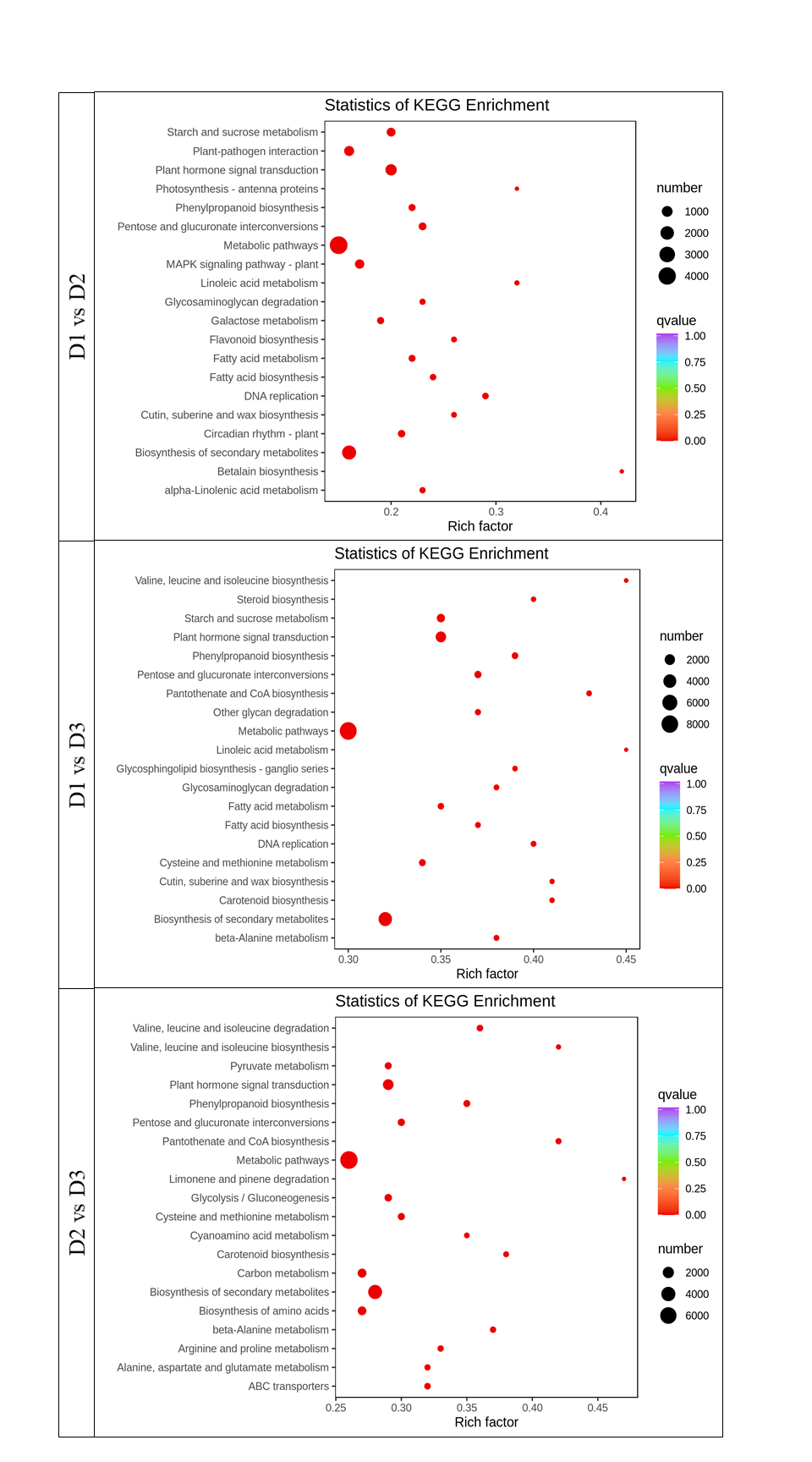

Supplement: Supplementary file 3 [file Image4.TIF]

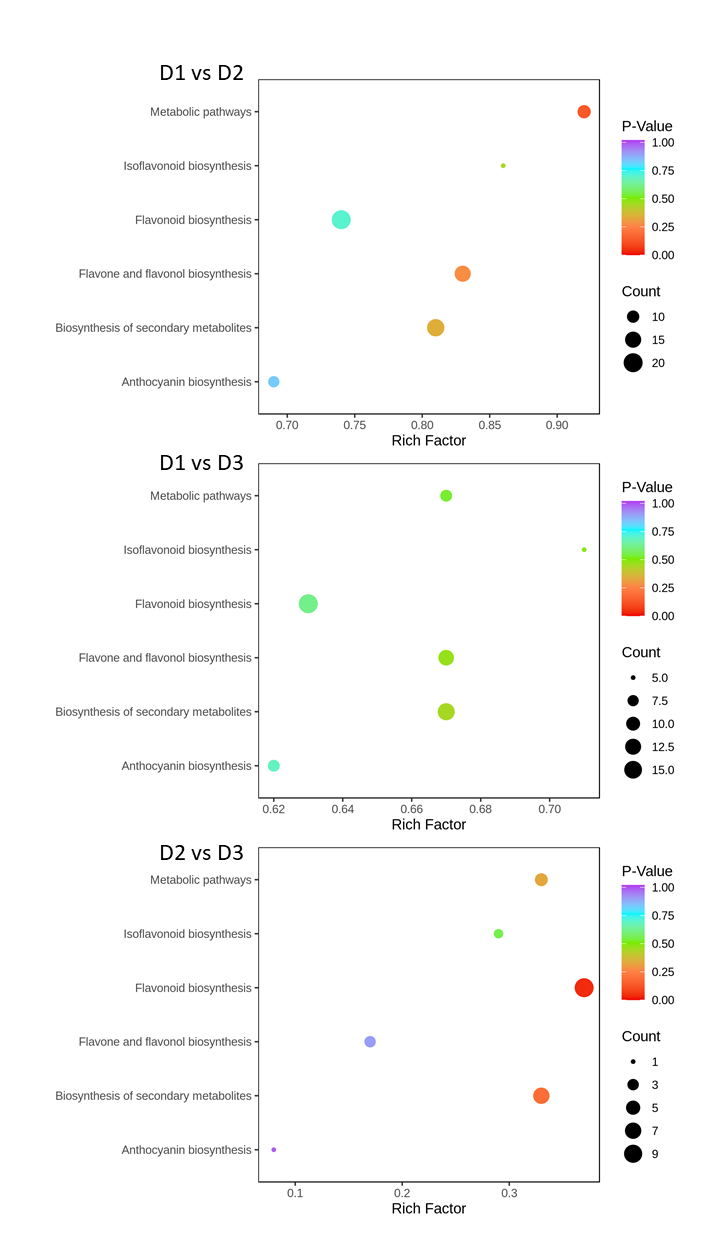

Supplement: Supplementary file 4 [file Image2.TIF]

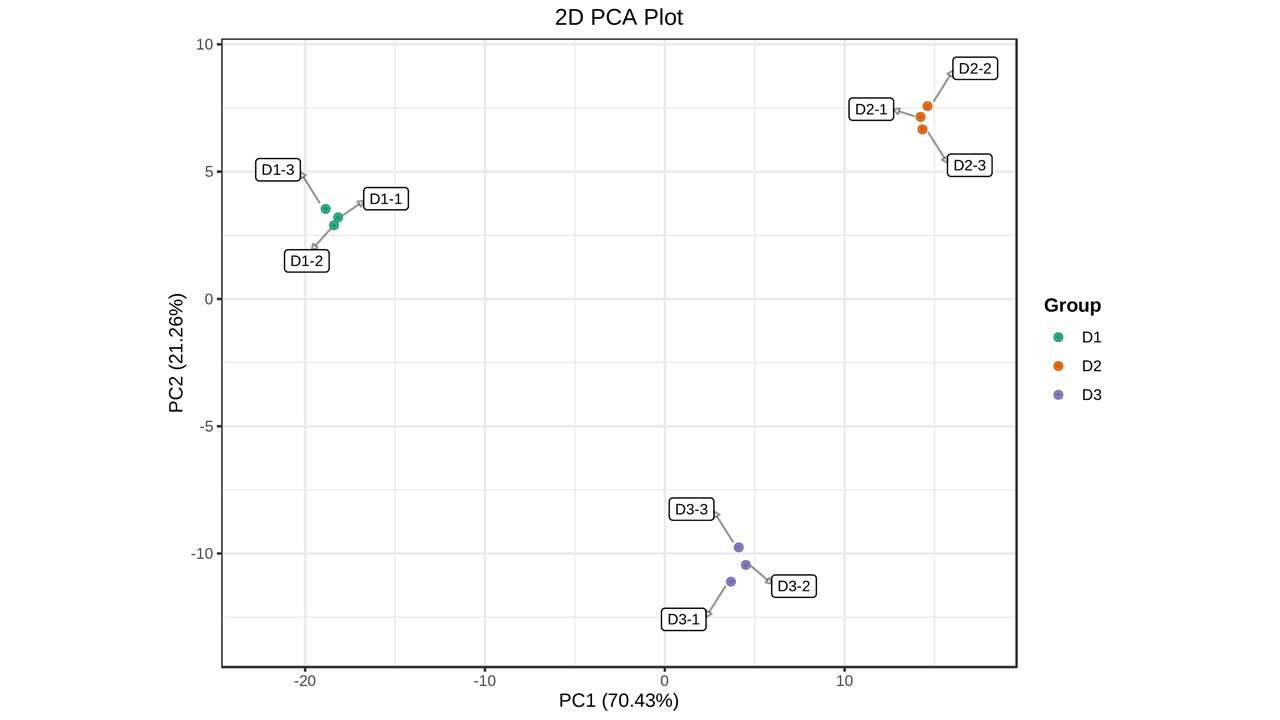

Supplement: Supplementary file 5 [file Image1.TIF]

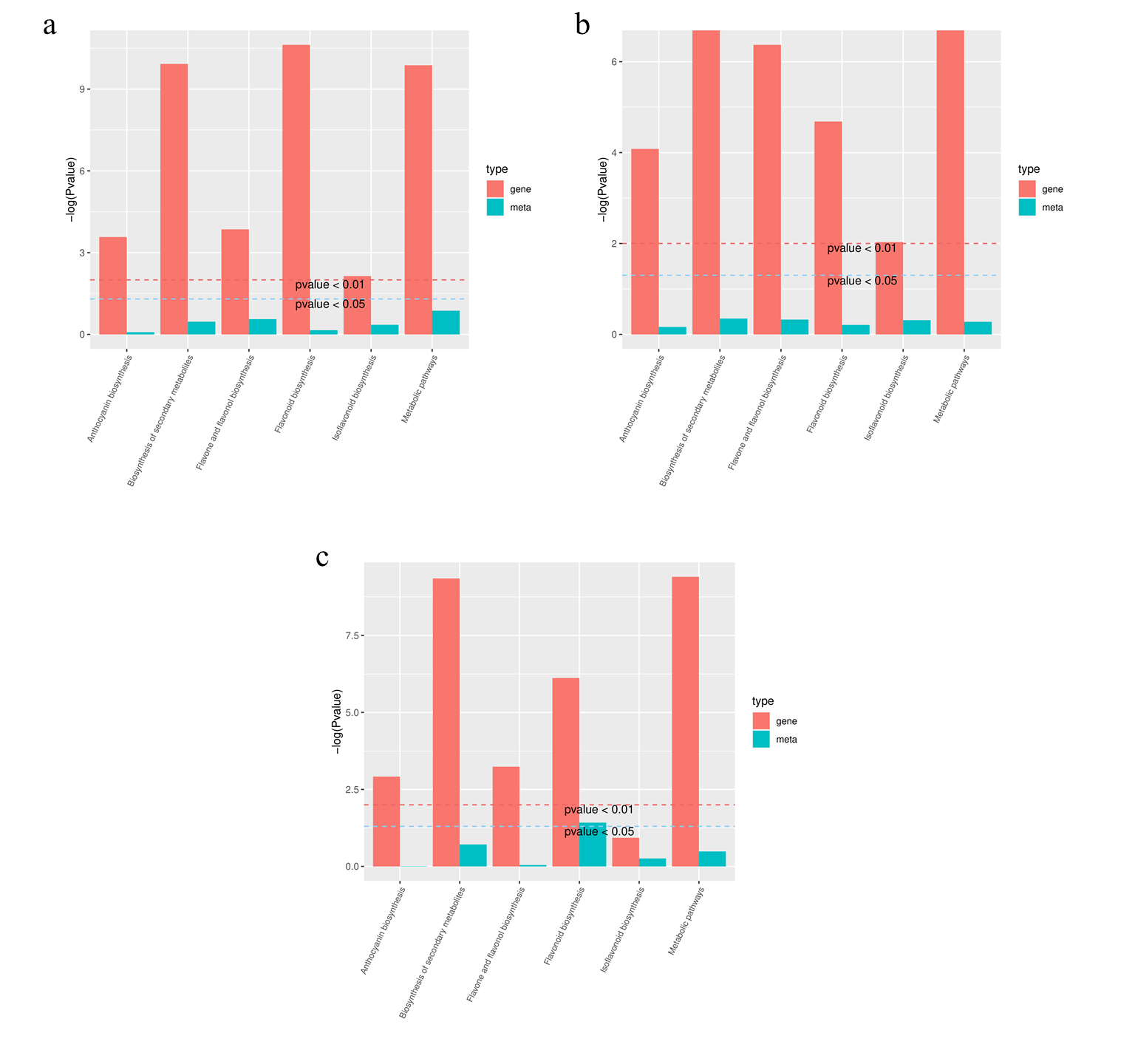

Supplement: Supplementary file 6 [file Image5.TIF]
